# Efficacy of radiotherapy in patients on progression after treatment with ipilimumab 3 mg/kg

**DOI:** 10.1186/1479-5876-12-S1-O6

**Published:** 2014-05-06

**Authors:** Antonio M Grimaldi, Ester Simeone, Diana Giannarelli, Paolo Muto, Sara Falivene, Fabio Sandomenico, Antonella Petrillo, Marcello Curvietto, Assunta Esposito, Miriam Paone, Marco Palla, Corrado Caracò, Gennaro Ciliberto, Nicola Mozzillo, Paolo A Ascierto

**Affiliations:** 1 Istituto Nazionale Tumori, Fondazione “G. Pascale”, Naples, Italy

## Background

Ipilimumab, a fully human monoclonal antibody (IgG1) that promote antitumor immunity by blocking CTLA4, was the first agent which showed a long-term survival benefit, about the 20% of patients, for the treatment of metastatic melanoma. The combination of ipilimumab with other therapies might improve its efficacy.

The term “abscopal effect” refers to a regression of metastatic lesions distant from the primary site of radiotherapy (RT). This new phenomenon represent the systemic response observed in patients who received ipilimumab.

Here we reported the outcomes from patients treated in the ipilimumab Italian expanded access program (EAP) who received RT after ipilimumab progression.

## Patients and Methods

Patients with advanced melanoma after ipilimumab progression were selected for analysis. Patients, who failed ipilimumab therapy and for whom no other therapeutic options were available, were elegible for radiotherapy.

## Results

21 out of 95 patients treated with ipilimumab in the Italian EAP were eligible for the analysis. The median age was of 58 years (range 21-77); The progression free survival (PFS) from ipilimumab treatment was 4 months (range 3-6), while the time from the end of treatment with ipilimumab and RT was of 5 months (range 4-8).

RT was performed on brain in 13 (62%) patients: 8 were treated with whole-brain RT and 5 patients with stereotactic RT. Other RT treatment included bone, metastatic distant lymph nodes, sub-cutaneous metastasis, spinal cord metastatis. The median doses of radiation was of 30 Gy (range 30-50). A local response to RT was detected in 13 patients (62%) while 8 patients (38%) did not show any local regression. The abscopal response has been detected in 11/21 (52%) patients: in details, we observed 9 abscopal partial response (42,8%), 2 abscopal stable disease (9,6%), and 10 progression (47,6%). The median of occurrence of the abscopal response was of 1 month (range 1-4). The median overall survival (OS) for all the 21 patients was of 13 months (range 6-26). The median OS for patients with and without abscopal responses was respectively of 22.4 months (range 2,5-50,3) and 8,3 months (range 7,6-9.0). 11 (84,6%) out of 13 patients with local response showed an abscopal effect.

## Conclusions

RT could be a new treatment option after ipilimumab treatment for potentiate its effect. Local response to RT might be predictive for the abscopal response and outcome. Further studies are warranted in this field to better understand and define the role of RT in combination or sequencing with ipilimumab treatment.

